# Molecular characterization and determination of the biochemical properties of cathepsin L of *Trichinella spiralis*

**DOI:** 10.1186/s13567-022-01065-6

**Published:** 2022-06-23

**Authors:** Ruo Dan Liu, Xiang Yu Meng, Chen Le Li, Shao Rong Long, Jing Cui, Zhong Quan Wang

**Affiliations:** Department of Parasitology, Medical College, Zhengzhou University, Zhengzhou, 450052 China

**Keywords:** *Trichinella spiralis*, cathepsin L, cysteine protease, enzymatic characterization, inhibitor

## Abstract

Cathepsin L is an important cysteine protease, but its function in *T. spiralis* remains unclear. The aim of this research was to explore the biological characteristics of *T. spiralis* cathepsin L (TsCatL) and its role in *T. spiralis*-host interactions. Bioinformatic analysis revealed the presence of the cysteine protease active site residues Gln, Cys, His and Asn in mature TsCatL, as well as specific motifs of cathepsin L similar to ERFNIN and GYLND in the prepeptide of TsCatL. Molecular docking of mature TsCatL and E64 revealed hydrophobic effects and hydrogen bonding interactions. Two domains of TsCatL (TsCatL2) were cloned and expressed, and recombinant TsCatL2 (rTsCatL2) was autocatalytically cleaved under acidic conditions to form mature TsCatL. TsCatL was transcribed and expressed in larvae and adults and located in the stichosome, gut and embryo. Enzyme kinetic tests showed that rTsCatL2 degraded the substrate Z-Phe-Arg-AMC under acidic conditions, which was inhibited by E64 and PMSF and enhanced by EDTA, L-cysteine and DTT. The kinetic parameters of rTsCatL2 were a Km value of 48.82 μM and Vmax of 374.4 nM/min at pH 4.5, 37 °C and 5 mM DTT. In addition, it was shown that rTsCatL2 degraded haemoglobin, serum albumin, immunoglobulins (mouse IgG, human IgG and IgM) and extracellular matrix components (fibronectin, collagen I and laminin). The proteolytic activity of rTsCatL2 was host specific and significantly inhibited by E64. rTsCatL2 possesses the natural activity of a sulfhydryl-containing cysteine protease, and TsCatL is an important digestive enzyme that seems to be important for the nutrient acquisition, immune evasion and invasion of *Trichinella* in the host.

**Supplementary Information:**

The online version contains supplementary material available at 10.1186/s13567-022-01065-6.

## Introduction

Trichinellosis, a worldwide foodborne zoonotic parasitosis, is caused by the tissue-dwelling nematode *Trichinella* spp. [1]. Human trichinellosis is primarily caused by the consumption of undercooked meat containing *Trichinella spiralis* muscle larvae (MLs). Outbreaks of human trichinellosis have been discovered in 55 countries worldwide [2]. A total of 65 818 cases of human trichinellosis were reported from 1986 to 2009 [3]. Trichinellosis is defined as an emerging or a re-emerging zoonotic parasitic disease, specifically in developing countries [4, 5]. When meat contaminated with *T. spiralis* MLs is ingested, the MLs are released from the capsule by the action of digestive juices. The larvae are activated to the intestinal infective larvae (IILs) form when peristalsis of the gastrointestinal tract reaches the small intestine, where they then invade the intestinal mucosa and develop into adult worms (AWs) within 48 h. Fertilized females produce newborn larvae (NBLs), which invade small veins or lymphatic vessels to reach all parts of the body, and larvae that reach the skeletal muscle continue to develop into MLs. The process of development and survival of the worms involves complex host-parasite interactions [6, 7]. Proteases of *T. spiralis* are indispensable in establishing parasitism and evading the host’s immune killing [8, 9]. Serine protease and aspartate protease promote the invasion of *T. spiralis* into the intestinal epithelium [10, 11]. *Trichinella* serine protease inhibitor can trigger anti-inflammatory mechanisms and regulate alternative activation of macrophages [12].

Cathepsin L, an important cysteine protease, is crucial for the parasite [13, 14]. Cathepsin L can participate in nutrient uptake by catabolizing host proteins into absorbable peptides [15], facilitating parasite migration within the host by cleaving host proteins such as fibronectin, laminin and natural collagen [16, 17], inactivating host immune defences by cleaving immunoglobulins, and inhibiting Th1-cell immune responses in infected experimental animals, allowing the parasites to evade host immune responses [18]. Cathepsin L has been considered an important target for the prevention of parasitic infections and has been extensively studied in *Schistosoma hepatica*, *Schistosoma mansoni*, and *Taenia solium*, but limited studies have been conducted on *T. spiralis* cathepsin L [14, 19]. A previous study indicated that *T. spiralis* cathepsin L promotes larval invasion of hosts and is associated with worm development and female fertility [20]. Clarifying the function of cathepsin L is crucial for comprehending the biology of *T. spiralis* and will also provide a basis for the development of antitrichinellosis drugs and vaccines.

Previous research revealed an unstudied cysteine protease named *T. spiralis* cathepsin L (TsCatL) (GenBank no. KRY31298.1) in *T. spiralis* MLs and IILs by LC–MS/MS [21]. Since the function of this protein has not been investigated, in our study, we aimed to express TsCatL in vitro, characterize the biochemical properties of this protein, and explore the *T. spiralis*-host interactions.

## Materials and methods

### Parasites and animals

*T. spiralis* (ISS534) were passaged and maintained in BALB/c mice. SPF BALB/c mice were purchased from the Experimental Animal Center of Henan Province.

### Worm collection and protein preparation

MLs were collected from the muscle of mice infected with *T. spiralis* for approximately 42 days by the artificial digestion method [22]. The procedure was as follows: 1 g of mouse tissue corresponded to 30 mL of manual digestion (1% pepsin, 0.7% hydrochloric acid, 0.9% NaCl), which was shaken for 3~5 h at 43 °C. Thirty mice were gavaged with 3000, 1000 and 500 MLs in 3 groups and killed at 6 h, 48 h and 6 days post-infection to collect IILs, 2-d AWs and 6-d AWs from the intestine, respectively [23, 24]. The detailed collection procedure was to dissect the small intestine longitudinally and then cut it into small segments of 2–3 cm and incubate those segments in saline at 37 °C for 1–2 h. The worms in the intestine would voluntarily burrow out of the small intestine. NBLs were collected by incubating 6-d AWs in RPMI-1640 for 24 h. The worms were repeatedly freeze–thawed three times in liquid nitrogen-ice water, ground for 1 min with a high-speed grinder, sonicated for 10 min, and then centrifuged at 12 000 × *g* for 30 min at 4 °C. The supernatant was the natural crude protein [25].

### TsCatL sequence analysis

Based on the amino acid sequence of TsCatL, the cDNA sequence of this protein was found in the *Trichinella* genome sequence. The physicochemical properties of TsCatL were analysed using the ProtParam tool [26]. The structural domains of TsCatL were predicted utilizing SMART [27, 28]. The amino acid sequences of TsCatL structural domains were compared to the sequences of cathepsin L from other organisms through Clustal OmegaX [29]. A sequence logo was used to display the consensus sequences of cathepsin Ls by the Weblog 3 tool [30, 31]. The evolutionary relationships of TsCatL were assessed by constructing a phylogenetic tree based on the neighbour-joining method using MEGA 7 [32]. The GenBank accession numbers of cathepsin L were as follows: *Trichinella spiralis* (KRY31298.1), *Trichinella patagoniensis* (KRY14446.1), *Trichinella murrelli* (KRX43986.1), *Trichinella pseudospiralis* (KRX91582.1), *Trichinella nativa* (KRZ61475.1), *Trichinella nelsoni* (KRX23179.1), *Trichinella* T8 (KRZ88876.1), *Trichinella britovi* (KRY58680.1), *Trichinella* T6 (KRX80302.1), *Trichinella* T9 (KRX65498.1), *Trichinella zimbabwensis* (KRZ06301.1), *Haemonchus contortus* (AAF86584.1), *Caenorhabditis elegans* (CAB07275.1), *Trichinella papuae* (KRZ70556.1), *Fasciola hepatica* (AAB41670.2, AAC47721.1, AAF76330.1), *Fasciola gigantica* (AAD23996.1), *Taenia solium* (AAS00027.1), *Echinococcus multilocularis* (BAF02517.1), *Taenia pisiformis* (AEG19548.1), *Mus musculus* (AAD32136), and *Homo sapiens* (AAH12612). The tree was rooted by *Homo sapiens* and *Mus musculus*.

### Molecular modelling and evaluation

The online software SWISS-MODEL was applied to construct 3D molecular models of TsCatL and mature TsCatL, and the images were processed using the tertiary structure visualization software VMD. The constructed 3D molecular models were assessed and validated by applying the molecular structure model evaluation software Verify 3D, ERRAT, PROCHECK, PROVE and WHATCHECK integrated with SAVES V 6.0 [33–38].

### Molecular docking

Semiflexible docking with mature TsCatL to the small molecule E64 was performed using AutoDock Vina software [39]. The docking result of E64 in the active pocket of mature TsCatL protease was demonstrated in two dimensions by protein–ligand interaction profiler software [40] and in three dimensions using PyMOL.

### Cloning of the TsCatL2 gene

The cDNA of *T. spiralis* AWs was selected as the template for PCR amplification of the two TsCatL domains (TsCatL2). Specific primers for TsCatL2, including BamH I and Hind III sequences (underlined), were designed with Primer 5 (5′CGC GGA TCC TGG ATT ATT TAC AAA GAA ATA TAC GG-3′ and 5′-CCC AAG CTT TCA TAT AAT CGG ATA GCT GGC GAA T-3′). The recombinant pMAL-c2x/TsCatL2 was transformed into Rosetta-gami B (DE3) cells [41]. The expression of rTsCatL2 was induced at 25 °C and 1.0 mM IPTG for 6 h. The rTsCatL2 protein was purified using amylose resin and detected by SDS–PAGE [42].

### Production of a mouse polyclonal antibody

Ten BALB/c mice were immunized by subcutaneous injection of 20 μg of rTsCatL2 mixed with MONTANIDE™ ISA 61 VG 1:1 (v/v), with one booster immunization three weeks later [43, 44]. After another two-week interval, polyclonal antibodies against rTsCatL2 were obtained by collecting blood from mice.

### SDS–PAGE and western blotting

The natural crude proteins from *T. spiralis* MLs, IILs, AWs, NBLs and rTsCatL2 were separated by SDS–PAGE [45]. Then, they were transferred onto nitrocellulose membranes and blocked with 5% skim milk. The strips of membrane containing rTsCatL2 were incubated with infection serum, anti-rTsCatL2 mouse serum, or normal serum at a 1:100 dilution. After incubation with HRP-conjugated goat anti-mouse IgG (1:5000 dilution; Sangon Biotech, China), the colouration was processed with DAB (Solarbio, China) [24]. The strips containing *T. spiralis* different stage proteins were probed with the anti-rTsCatL2 antibody and processed with a chemiluminescent kit (Meilunbio, China) [11].

### Quantitative real-time PCR

The cDNAs of MLs, IILs, AWs, and NBLs were prepared according to previous references [9, 46]. Specific primers were designed by Primer 5 (5′-TACGGAAAAACGTATGCAAATG-3′; 5′-CAAATTCTCCATGAGTCAAATCGG-3′). GAPDH (GenBank: AF452239) was selected as the internal reference gene, as previously reported [23, 42]. The specific primers for GAPDH were as follows: 5′-AG ATGCTCCTATG TTGGTTATGGG-3′; 5′-GTCTTTTGGGTTGCCGTTGTAG-3′. qPCR was performed using SYBR Green qPCR Master Mix (TargetMol, China) on an Applied Biosystems 7500 qPCR machine. Subsequently, the relative transcript levels of TsCatL were analysed using the comparative Ct (2^−ΔΔCt^) method [46].

### Immunolocalization

A portion of intact parasites was fixed in prechilled acetone to detect whether TsCatL was expressed on the surface of *Trichinella*. In addition, another portion of worms was fixed in 4% formaldehyde and used to make paraffin sections to observe the localization of TsCatL in the worm tissue. All the samples were blocked with 5% goat serum, incubated at 4 °C overnight with anti-rTsCatL2 serum diluted at 1:100, and then incubated with FITC-conjugated goat anti-mouse IgG (1:100; Proteintech, USA). The paraffin sections were dyed with DAPI for 5 min and observed under a fluorescence microscope [47]. Preimmune serum and infection serum served as the negative control and positive control, respectively.

### Enzyme activity assay

The substrate Z-Phe-Arg-AMC (Sangon, Shanghai) was used to test the enzymatic activity of rTsCatL2 [48]. First, 2 μg/mL rTsCatL2 and 5 μM substrate were preincubated in 50 μL of assay buffer for 30 min, followed by mixing for 30 min under different conditions, and finally reaction termination solution (pH 4.5 HAc-NaAc buffer containing 0.1 M sodium chloroacetate) was added. The fluorescence intensity was analysed by using spectrophotofluorometry (Synergy H1, BioTek, USA) at an excitation wavelength of 355 nm and an emission wavelength of 460 nm [48]. The effect of temperature on rTsCatL2 activity was assayed at 35 ℃, 40 ℃, 45 ℃, 50 ℃, 55 ℃, 60 ℃, 65 ℃ and 70 ℃. The optimal pH for rTsCatL2 activity was assayed using assay buffers with different pH values: 100 mM Gly-HCl buffer (pH 2.0–3.0), 100 mM HAc-NaAc (pH 3.5–5.5), 100 mM Na_2_HPO_4_-NaH_2_PO_4_ (pH 6.0–7.5) and 100 mM Tris–HCl (pH 8.0). The effect of metal ions on the relative enzyme activity of rTsCatL2 was assessed by adding different concentrations (0.1, 5, 50 and 100 mM) of Mn^2+^, Cu^2+^, Mg^2+^, Ni^2+^ and Zn^2+^ to the assay buffer. Inhibitors (10 μM E64, 1 mM PMSF, 1 mM AEBSF, 1 mM 1, 10-phenanthroline and 1 mM pepstatin A) and agonists (1 mM EDTA, 1 mM L-Cys and 1 mM DTT) were preincubated with rTsCatL2, and the effect on rTsCatL2 enzyme activity was assessed. The kinetic parameters of rTsCatL2 for Z-Phe-Arg-AMC were also determined.

### Degradation of different proteins by rTsCatL2

The natural substrate proteins tested include haemoglobin (Hb), serum albumin, immunoglobulin (Ig), fibronectin, collagen I and laminin. Haemoglobin (Hb) from mice, humans, swines, bovines and chickens was acquired from fresh erythrocytes [49]. Bovine serum albumin (BSA), human serum albumin (HSA), human IgG, human IgM and mouse IgG, fibronectin, collagen I and laminin were purchased from Sigma. First, 20 μg of substrate proteins were incubated with 0.5 μg of rTsCatL2 in a pH 3–6 buffer solution overnight, and then hydrolysates were detected by SDS–PAGE. Each protein was also incubated with 2 μg of MBP at 37 °C overnight as a control. The inhibitor (10 μM E64) was incubated with rTsCatL2 for 30 min and then incubated with each protein overnight to detect protein degradation.

### Statistical analysis

The data were statistically analysed by SPSS 21.0 and are expressed as the arithmetic mean ± standard deviation. Differences in TsCatL2 mRNA transcription were analysed by one-way ANOVA.

## Results

### Bioinformatic analysis of TsCatL

**Figure 1:**
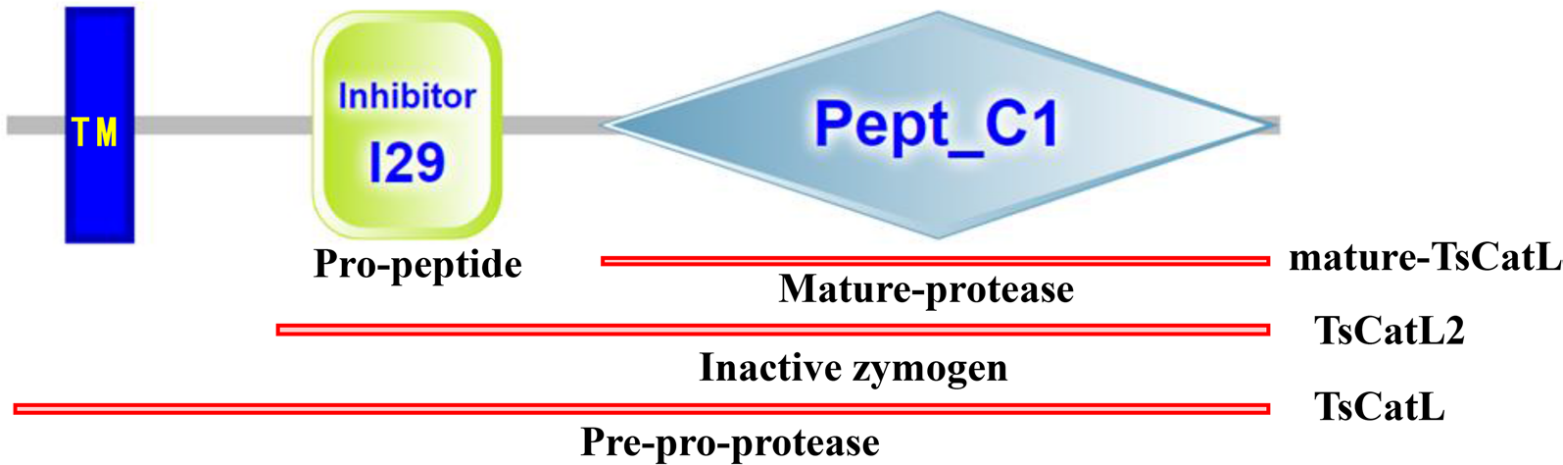
**Domain organization of TsCatL.**

**Figure 2:**
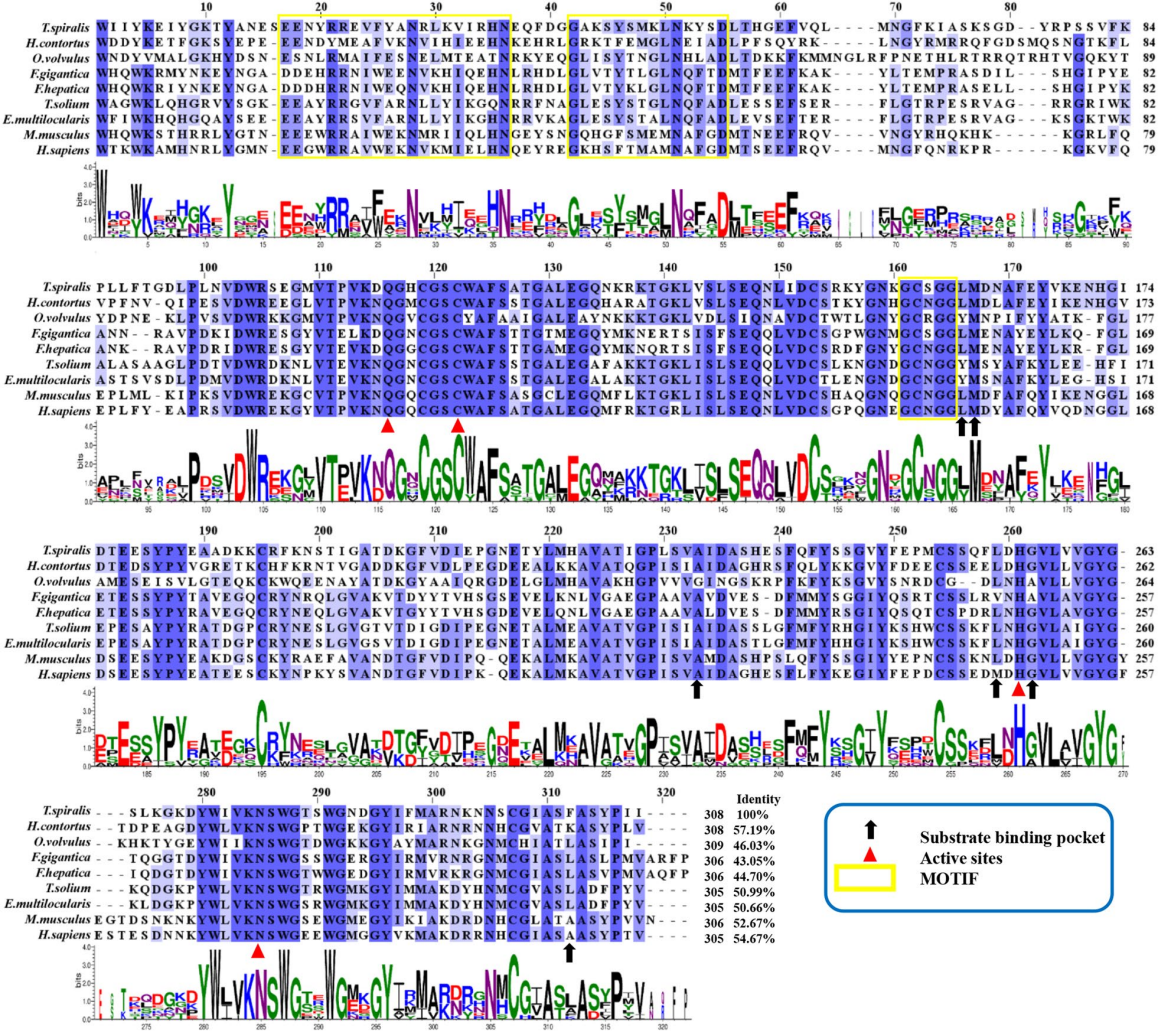
**Multiple alignment and sequence logo identification of cathepsin L.** Identical and similar residues are labelled with blue shading. Putative active site residues (Gln [Q], Cys [C], His [H] and Asn [N]) are labelled with red triangles. Substrate-binding pocket residues (Leu [L], Met [M], Ala [A], Leu [L], Gly [G] and Phe [F]) are labelled with black arrows, and the ERFNIN, GYLND and GCN/SGG motifs are labelled with yellow boxes.

**Figure 3:**
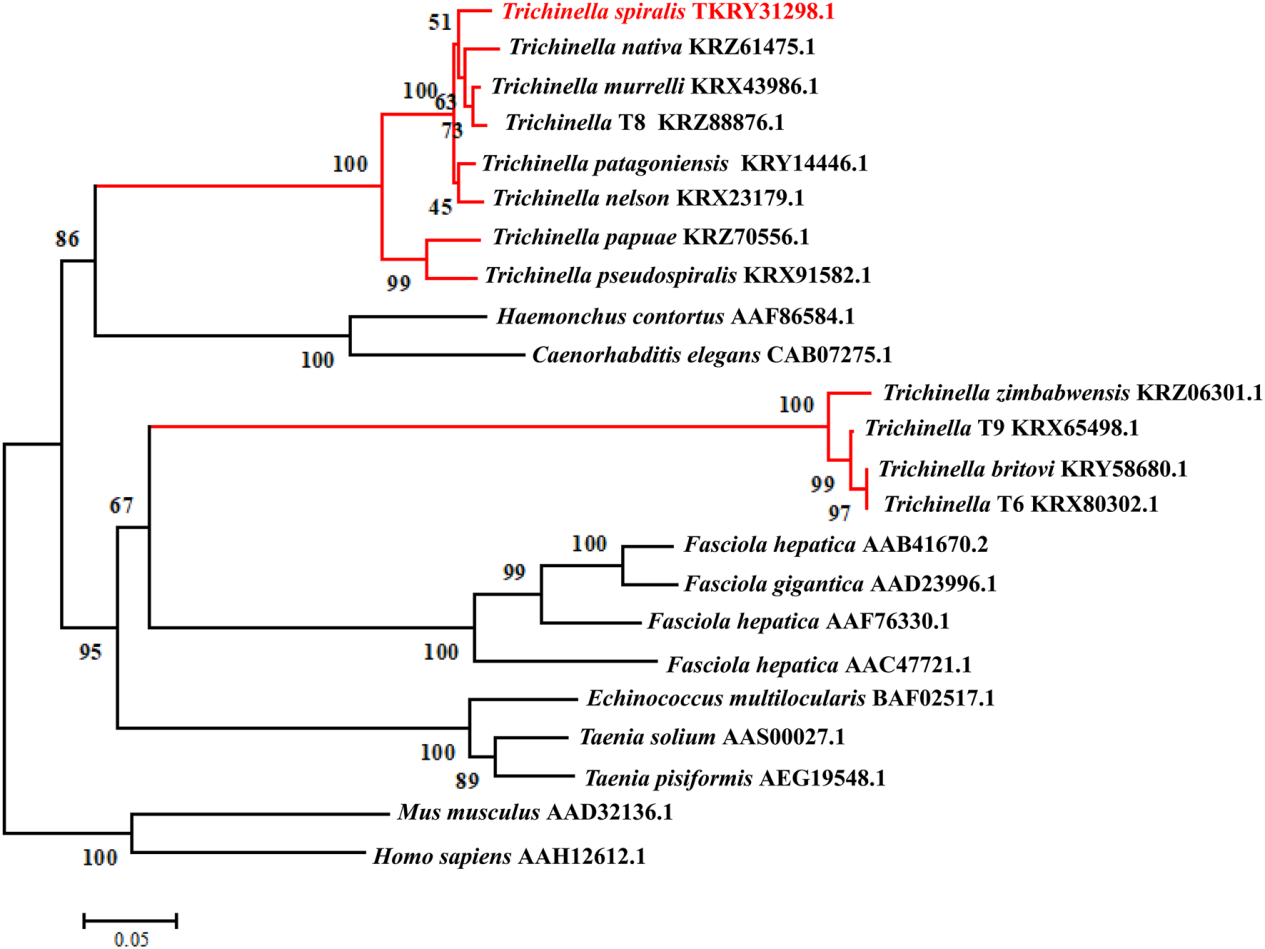
**Phylogenetic analysis of cathepsin L family proteins.**

TsCatL consisted of 1356 bp and encoded 404 amino acid residues, with an MW of 45.59 kDa and an isoelectric point of 8.27. SMART analysis showed that the TsCatL protein contained a transmembrane helix (amino acids 20–39) at the N-terminus, an inhibitor_I29 domain (amino acids 97–157) and a mature Pept_C1 domain (amino acids 188–403). The inhibitor_I29 domain is a prepeptide, the Pept_C1 domain is a mature peptide, and the two structural domains of *T. spiralis* cathepsin L were named TsCatL2 in this study (Figure 1). Sequence alignment showed that the amino acid sequence identity of TsCatL with other cathepsin L proteins was higher than 40%. TsCatL was predicted to be a typical cathepsin L-like cysteine peptidase with conserved cysteine protease active site residues (Gln, Cys, His and Asn), substrate-binding pocket residues (Leu, Met, Ala, Leu, Gly and Phe) and a cathepsin L-specific motif (ERFNIN-like ERFNVN, GYLND and GCN/SGG) (Figure 2). A phylogenetic tree showed that the *Trichinella* genus has two clades, and TsCatL has the closest evolutionary relationship with *T. native*, T8 and *T. murrelli* (Figure 3).

### Molecular modelling and evaluation

**Figure 4:**
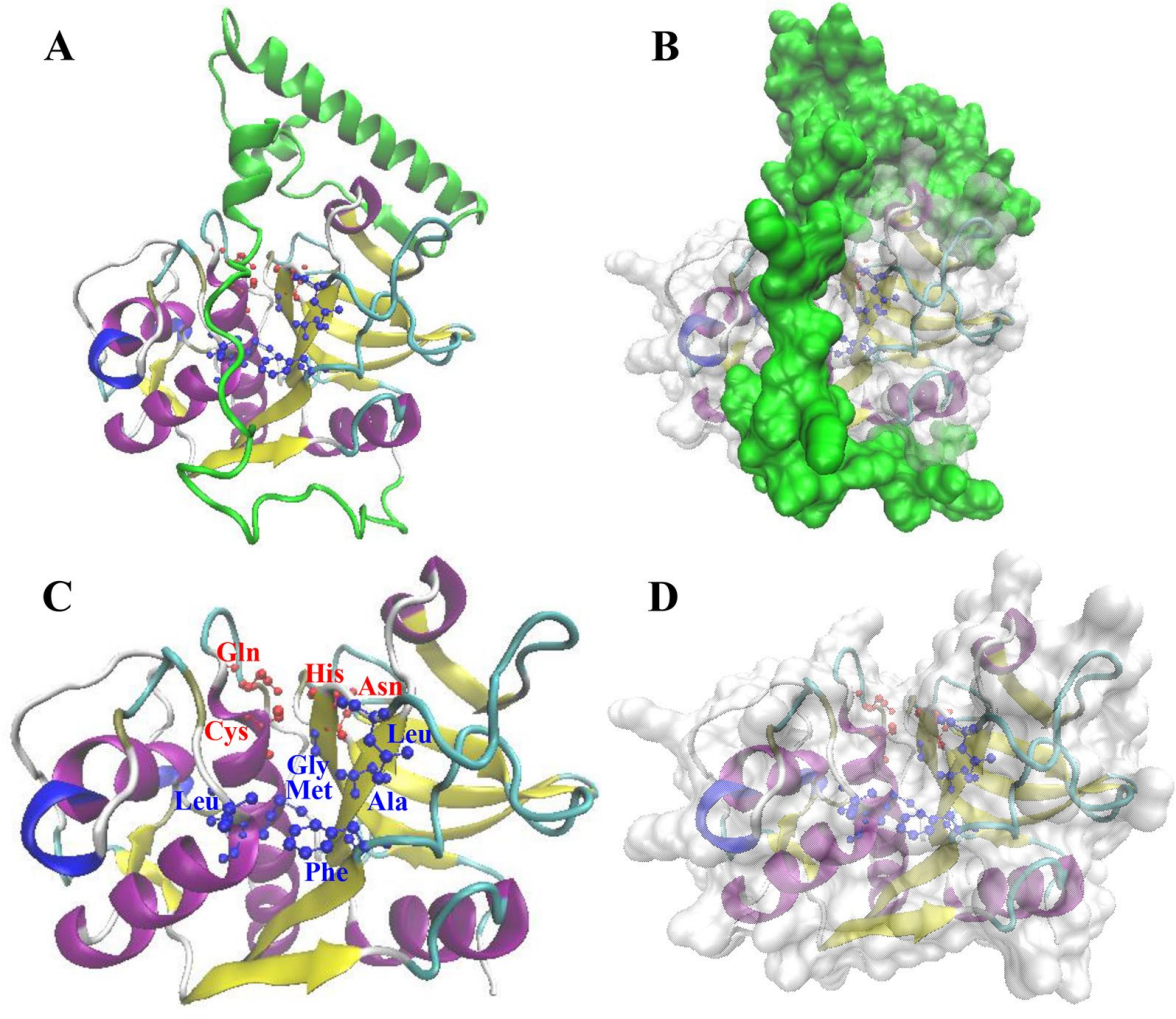
**Predicted three-dimensional structures of TsCatL2 and mature TsCatL.** The propeptide (green colour) blocks the active sites and substrate-binding pocket of the protease to prevent substrate exposure. The active sites of TsCatL are marked in red, and the substrate binding pocket is marked in blue.

The 3D structures of the inactive zymogen of TsCatL2 and mature TsCatL were predicted using the crystal structure of human cathepsin L (PDB ID: 6JD0) as the template by SWISS-MODEL. The TsCatL2 and mature TsCatL 3D models were further tested by SAVES v5.0. In the 3D-1D profile, 85.06% of TsCatL2 residues and 90% of mature TsCatL residues had a score of ≥ 0.2. The overall quality factors of TsCatL2 and mature TsCatL were 91.639 and 94.203, respectively, according to the ERRAT results. The Ramachandran plot revealed that all TsCatL2 and mature TsCatL residues were in the most favoured or disallowed regions (Additional files 1 and 2). Both models also passed the WHATCHECK, PROVE and PROCHECK assessments. In the TsCatL2 model, the propeptide (green colour) blocks the active sites and substrate-binding pocket of the protease to prevent substrate exposure (Figures 4A, B). In the mature TsCatL model, the left domain contains three α-helix motifs, and the right domain primarily consists of β-folded sheets. The cleft containing the active sites is localized at the junction of the two domains (Figures 4C, D).

### Molecular docking between mature TsCatL and E64

**Figure 5:**
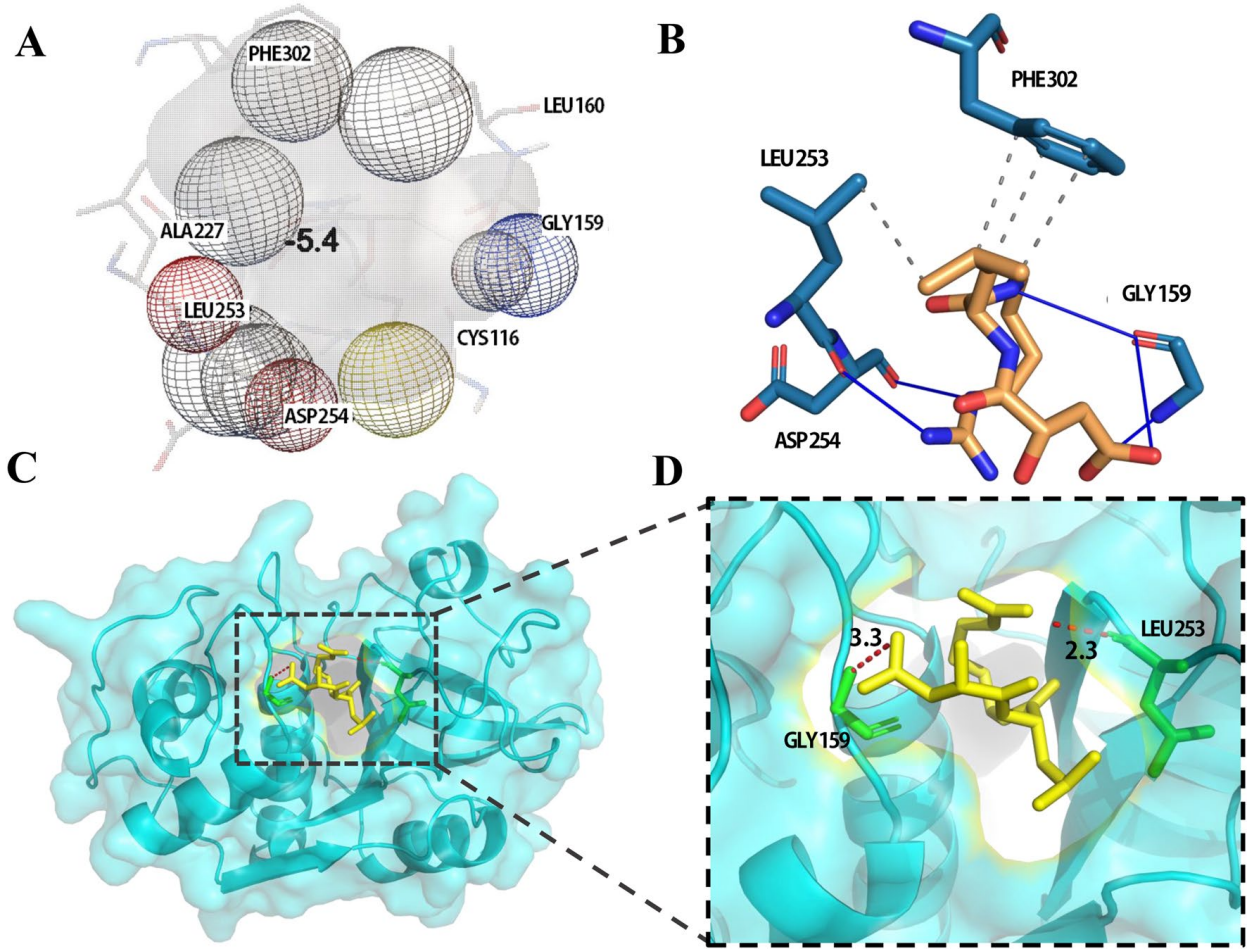
**Stereo view of the interactions between mature TsCatL and E64.** **A** The result of AutoDock Vina analysis; **B** two-dimensional display of the docking results produced by the protein–ligand interaction profiler. The grey dashed lines represent hydrophobic interactions, and the blue solid lines represent hydrogen bonds. **C**, **D** Docking results created with PyMOL. TsCatL Leu and Gly residues are further indicated in green stick representation, E64 is shown in yellow stick representation, and red dotted lines represent hydrogen bonds.

AutoDock Vina was used to calculate the affinity between mature TsCatL and E64 based on the efficient optimization algorithm of the scoring function and select 9 binding conformations. According to the binding affinity and binding site analysis, the lowest binding free energy (-5.4 kcal/mol) model was selected. In the mature TsCatL-E64 complex, E64 was located in a pocket formed by 7 amino acid residues (Cys, Gly, Leu, Ala, Leu, Asp and Phe) (Figure 5A). Using the online protein–ligand interaction profiler software to analyse the docking results, it was found that the mature TsCatL-E64 complex formed hydrophobic interactions with Leu253 (~3.72 Å) and Phe302 (~3.68 Å) and hydrogen bonding with Gly159 (~3.33 Å), Leu253 (~2.31 Å), and Asp254 (~2.21 Å) (Figure 5B). PyMOL analysis revealed hydrogen bonding interactions with Gly159 (~3.3 Å) and Leu253 (~2.3 Å) (Figures 5C, D).

### Expression of rTsCatL2 and Western blot analysis

**Figure 6:**
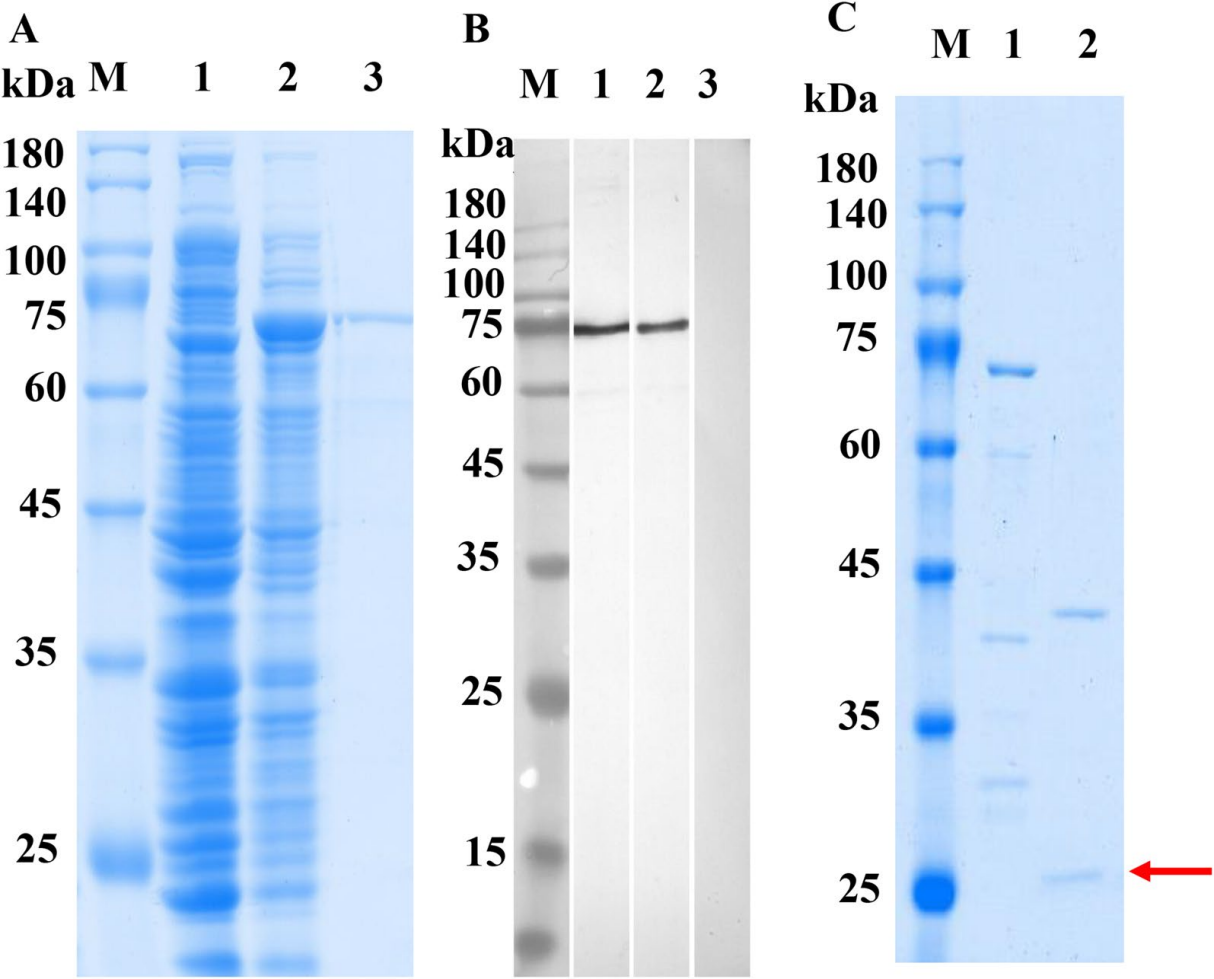
**Identification of rTsCatL2.** **A** SDS–PAGE. Lane 1: recombinant bacterial pMAL-c2x-TsCatL2 lysate before induction; Lane 2: pMAL-c2x-TsCataL2 lysate after 1 mM IPTG induction at 25 °C; Lane 3: purified rTsCatL2 with MBP. **B** Western blotting analysis of rTsCatL2 antigenicity. Lane 1: Serum of mice infected with *T. spiralis*; Lane 2: anti-rTsCatL2 serum; Lane 3: serum of normal mice. **C** Analysis of autocatalytic cleavage of rTsCatL2 at 37 °C for 30 min. Lane 1: pH 7.0; Lane 2: pH 5.0. Mature-TsCatL is indicated with a red arrow.

**Figure 7:**
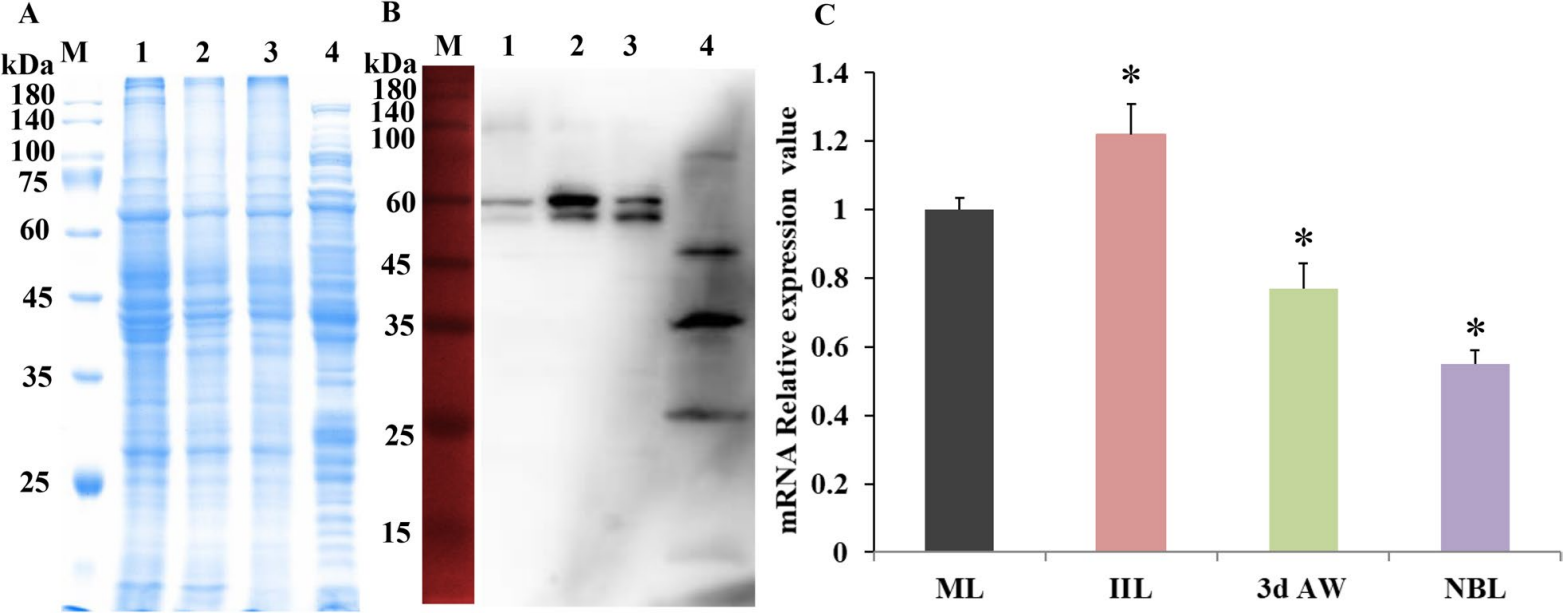
**TsCatL expression and transcription in T. spiralis.** **A** SDS–PAGE of *T. spiralis* soluble proteins; Lane 1: MLs, Lane 2: IILs, Lane 3: AWs, Lane 4: NBLs. **B** Western blotting analysis of TsCatL in crude proteins. Lane 1: MLs, Lane 2: IILs, Lane 3: AWs, Lane 4: NBLs. The proteins were recognized by anti-rTsCatL2 serum. **C** TsCatL mRNA expression levels (*P < 0.001).

The 927-bp CDS of TsCatL2 was amplified, which encodes 308 amino acids from 97 to 404, including the inhibitor_I29 domain and Pept_C1 domain. The SDS–PAGE results showed that the MW of rTsCatL2 was 71 kDa (containing the 43 kDa MBP tag) after purification (Figure 6A). The Western blot results showed that rTsCatL2 was identified by infection sera and anti-rTsCatL2 sera but not by sera of normal mice (Figure 6B). After incubation at pH 5.0 for 30 min, rTsCatL2 formed mature TsCatL with an approximate molecular weight of 27 kDa by autocatalytic cleavage (Figure 6C). Using anti-rTsCatL2 serum, protein bands of 60 and 56 kDa were identified in *T. spiralis* MLs, IILs and 2-d AWs, but four protein bands (approximately 85, 49, 37, and 27 kDa) were identified in NBLs (Figures 7A, B). qPCR analysis indicated that the mRNA transcription of TsCatL was highest in IILs and lowest in NBLs (F = 126.483, *P* < 0.05) (Figure 7C).

### Immunolocalization

**Figure 8:**
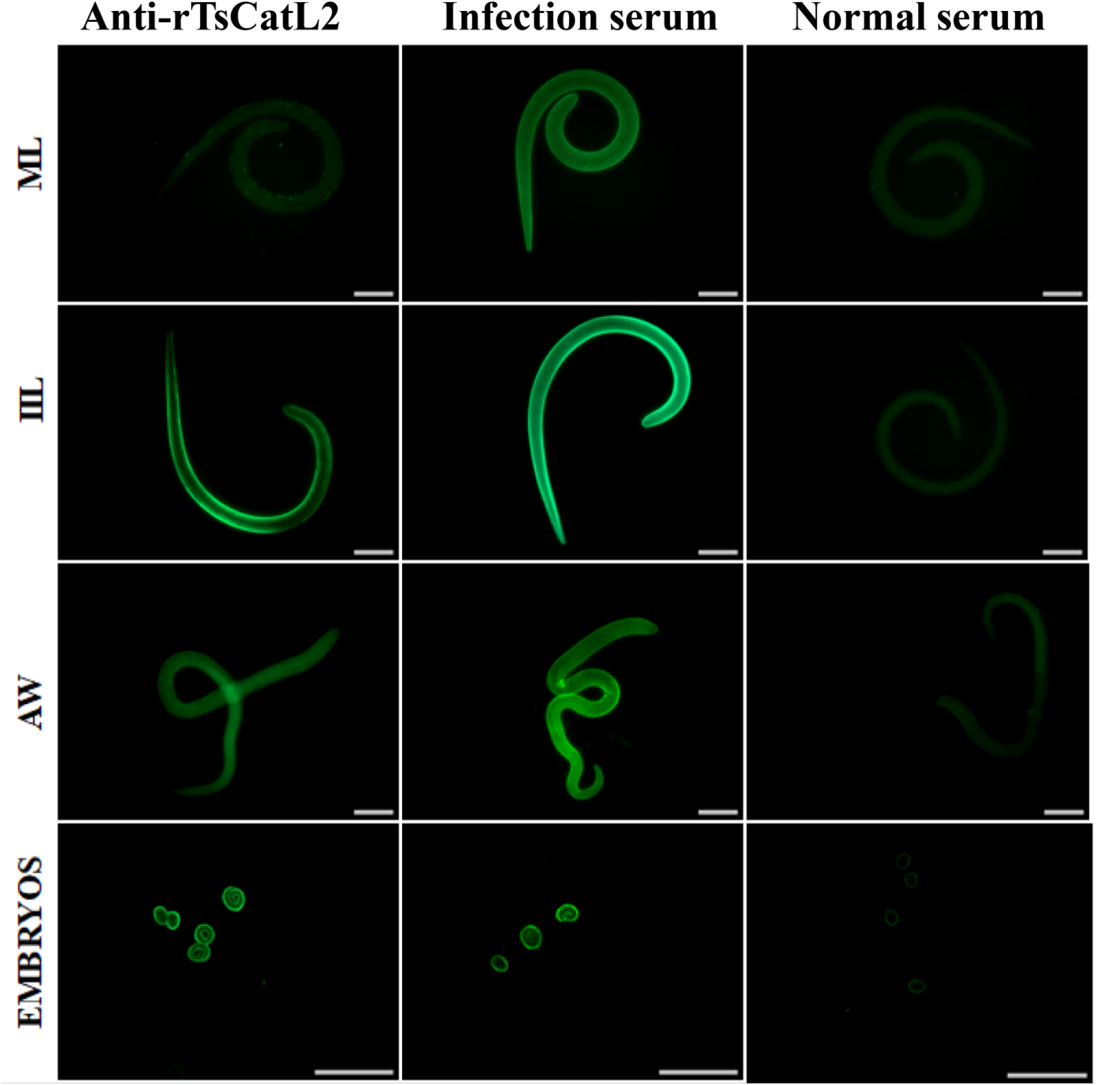
**Immunofluorescence detection of TsCatL on the surface of T. spiralis.** Intense green staining was detected on the cuticles of 6-h IILs, 2-d AWs and embryos by anti-rTsCatL2 serum. Preimmune serum and anti-*T. spiralis* serum were used as negative and positive controls, respectively. Scale bars: 100 μm.

**Figure 9:**
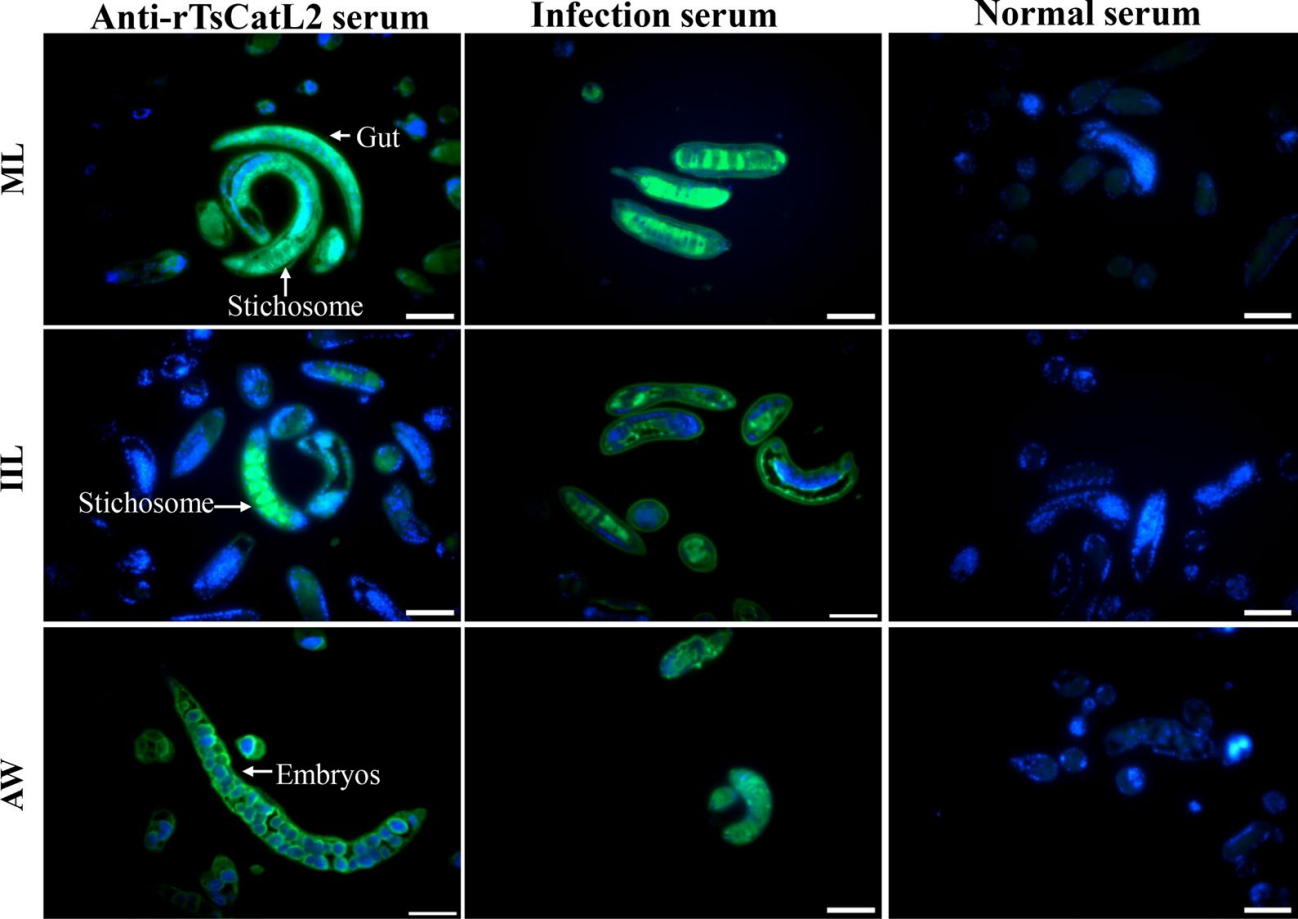
**Immunolocalization of TsCatL by IFA in paraffin sections of T. spiralis MLs, 6-h IILs and 6-d AWs.** Worm sections were tested by IFA. Using anti-rTsCatL2 sera, fluorescence was detected in the stichosome and gut of MLs and 6-h IILs, in addition to around embryos of female adult worms. Preimmune serum and anti-*T. spiralis* serum were used as negative and positive controls, respectively. The cell nuclei of worms were stained blue with DAPI. Scale bars: 50 μm.

Fluorescence detection of the whole worm indicated that intense green staining was marked on the cuticles of IILs, 2-d AWs and embryos of 6-d AWs (Figure 8). Fluorescence detection of paraffin sections showed that TsCatL was primarily located in the stichosome and gut of MLs and IILs, as well as embryos of adult worms (Figure 9).

### rTsCatL2 enzyme activity

**Figure 10:**
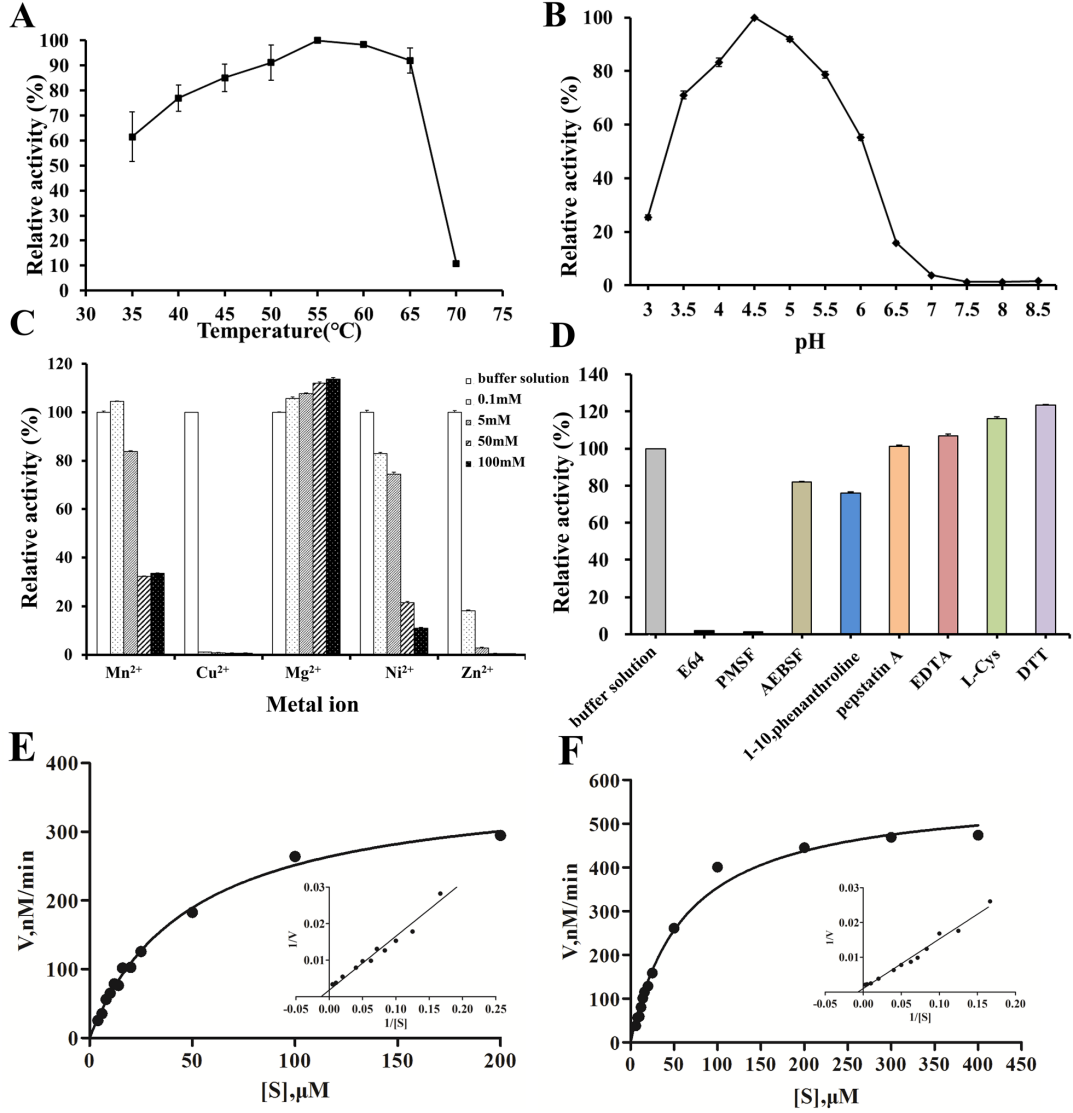
**Enzymatic characteristics of rTsCatL2.** **A** Different temperatures. **B** Different pH values at 55 °C. **C** Treatments with different metal ions. **D** Different inhibitors and activators. The concentration of E64 was 10 μM, and PMSF, AEBSF, 1,10-phenanthroline, pepstatin A, EDTA, L-cysteine and DTT were used all at 1 mM. **E** Michaelis–Menten curve and Lineweaver–Burk plot of rTsCatL2 at pH 4.5, 37 °C and 5 mM DTT. **F** Michaelis–Menten curve and Lineweaver–Burk plot of rTsCatL2 at pH 4.5, 55 °C and 5 mM DTT.

The assay of rTsCatL2 enzyme activity was performed using the fluorescent substrate Z-Phe-Arg-AMC (Figure 10). The optimum temperature of rTsCatL2 activity was 55 °C. rTsCatL2 exhibited enzymatic activity from pH 3 to 6.5, and the highest activity was pH 4.5 at 55 °C. Metal ions also affect the enzymatic activity of rTsCatL2. The activity was inhibited by Cu^2+^, Ni^2+^, and Zn^2+^ at concentrations of 0.1 mM, 5 mM, 50 mM and 100 mM and Mn^2+^ at concentrations of 5 mM, 50 mM and 100 mM. Conversely, the enzymatic activity of rTsCatL2 was enhanced by Mg^2+^ in a dose-dependent manner. In addition, the activity of rTsCatL2 was inhibited by E64 and PMSF and enhanced by EDTA, L-cysteine and DTT. However, pepstatin A had no effect on rTsCatL2 hydrolysis activity. Furthermore, the maximum hydrolytic velocity (Vmax) of rTsCatL2 was 374.4 nM/min, with a Michaelis constant (km) value of 48.82 μM at pH 4.5, 37 °C and 5 mM DTT. The Vmax of rTsCatL2 was 572.9 nM/min, with a km value of 61.96 μM at pH 4.5, 55 °C and 5 mM DTT.

### Cleavage of different proteins by rTsCatL2

**Figure 11:**
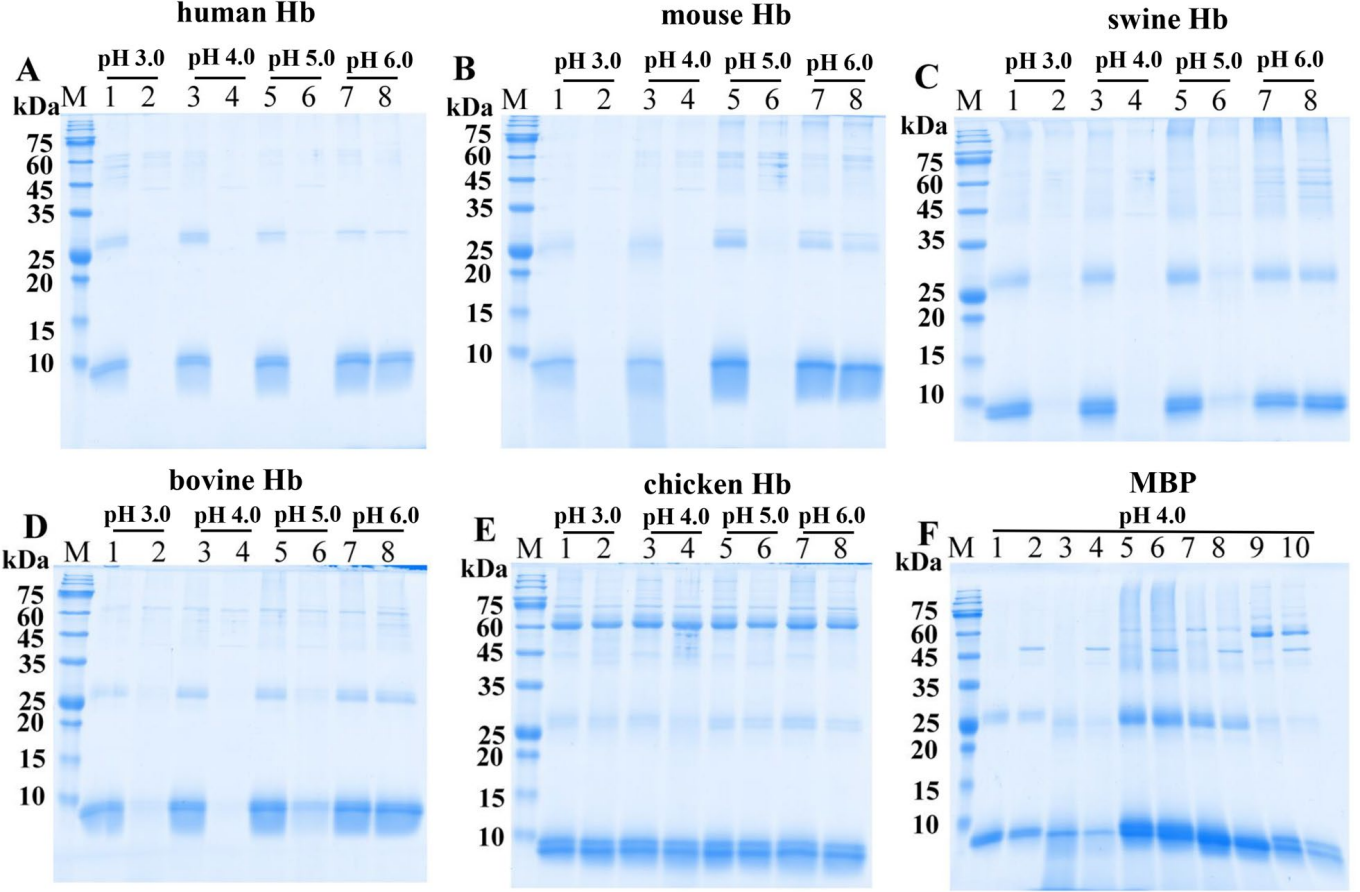
**Degradation of haemoglobin (Hb) from various hosts by rTsCatL2.** **A**–**E** Degradation of human Hb (**A**), mouse Hb (**B**), swine Hb (**C**), bovine Hb (**D**) and chicken Hb (**E**) by rTsCatL2 at different pH values. Lanes 1, 3, 5 and 7: Hb; Lanes 2, 4, 6 and 8: Hb + rTsCatL2; (F) Effect of MBP on hydrolysis of human Hb (Lanes 1–2), mouse Hb (Lanes 3–4), swine Hb (Lanes 5–6), bovine Hb (Lanes 7–8) and chicken Hb (Lanes 9–10) at pH 4.0. Lanes 1, 3, 5, 7 and 9: Hb; Lanes 2, 4, 6, 8 and 10: Hb + MBP.

**Figure 12:**
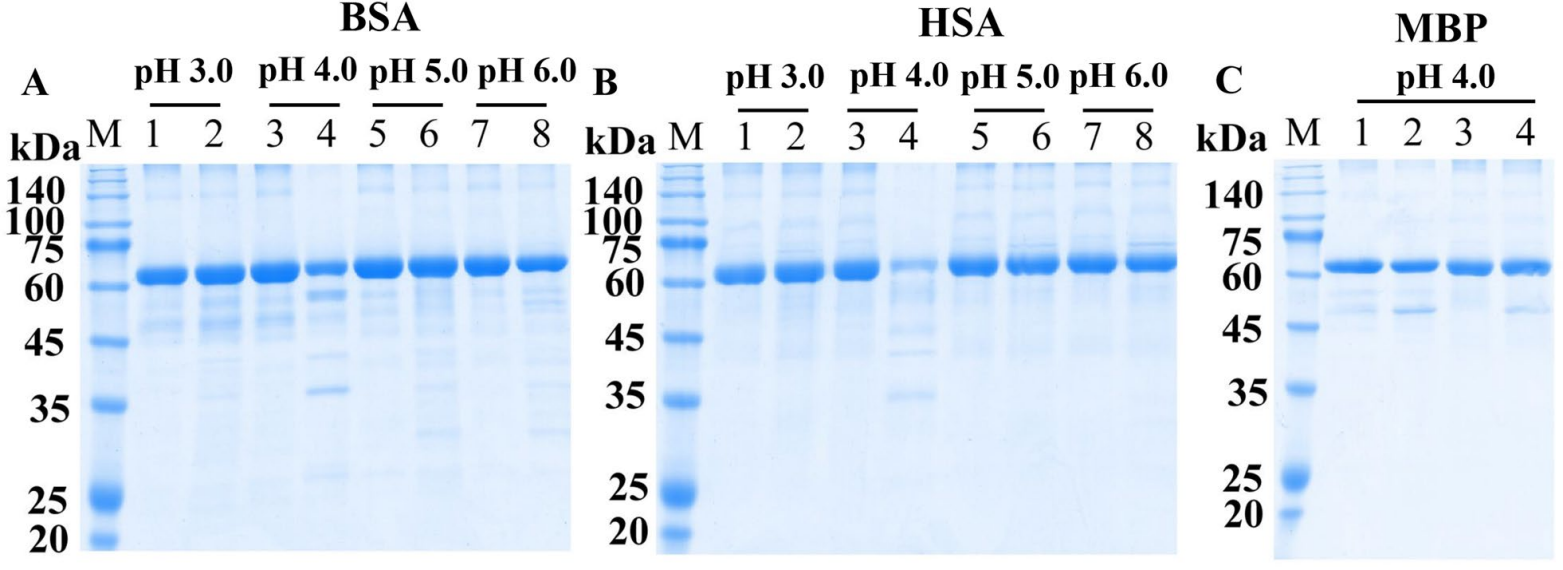
**Degradation of bovine serum albumin (BSA) and human serum albumin (HSA).** (**A**, **B**) Degradation of BSA (**A**) and HSA (**B**) by rTsCatL2 at different pH values. Lanes 1, 3, 5 and 7: serum albumin; Lanes 2, 4, 6 and 8: serum albumin + rTsCatL2. **C** Effect of MBP on hydrolysis at pH 4.0; Lane 1: BSA; Lane 2: BSA + MBP; Lane 3: HSA; Lane 4: HSA + MBP.

**Figure 13:**
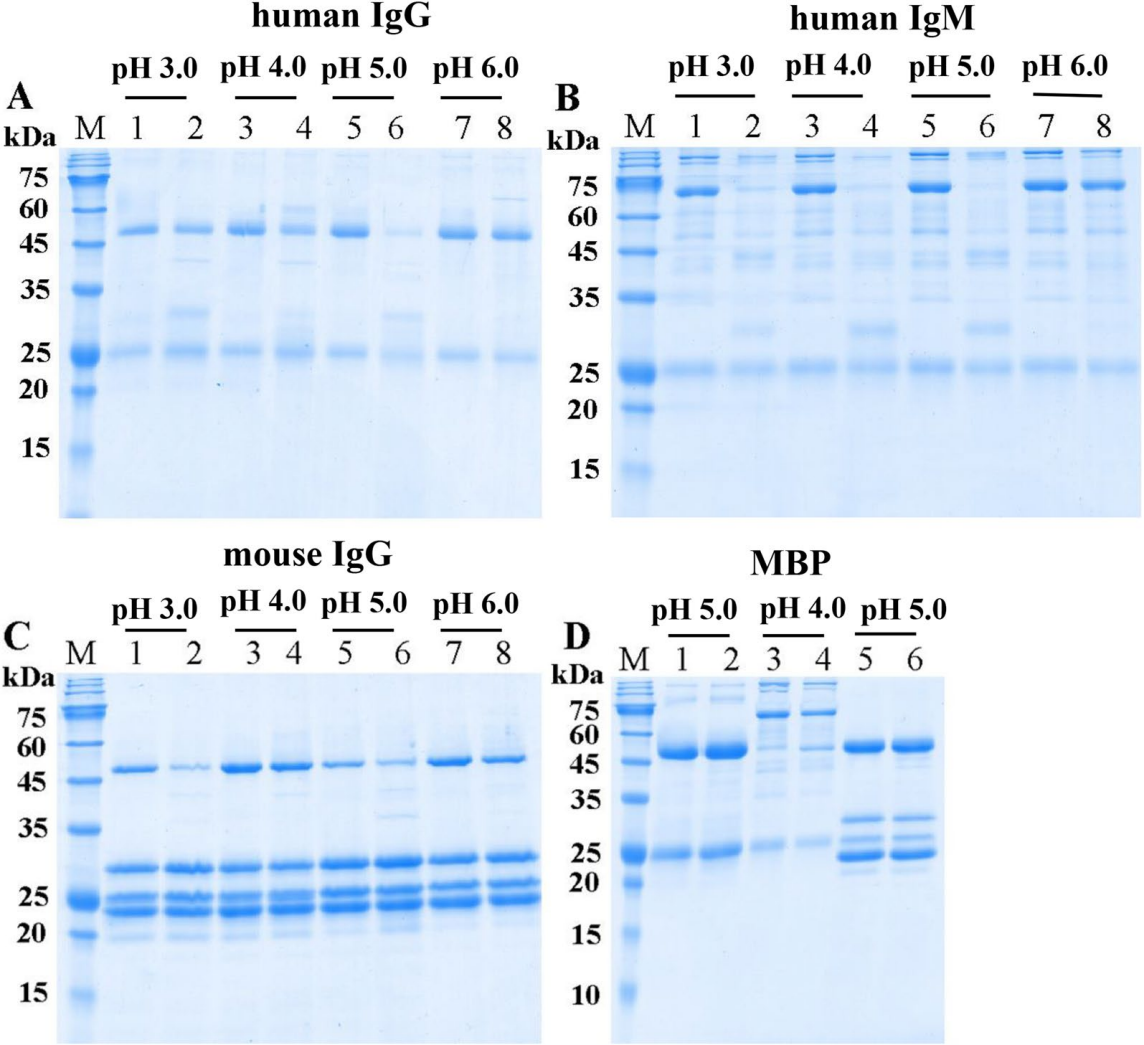
**Degradation of immunoglobulin.** **A**–**C** Hydrolysis of human IgG (**A**), human IgM (**B**) and mouse IgG (**C**) by rTsCatL2 at different pH values. Lanes 1, 3, 5 and 7: Ig; Lanes 2, 4, 6 and 8: Ig + rTsCatL2. **D** Effect of MBP on hydrolysis; Lane 1: human IgG; Lane 2: human IgG + MBP; Lane 3: human IgM; Lane 4: human IgM + MBP; Lane 5: mouse IgG; Lane 6: mouse IgG + MBP.

**Figure 14:**
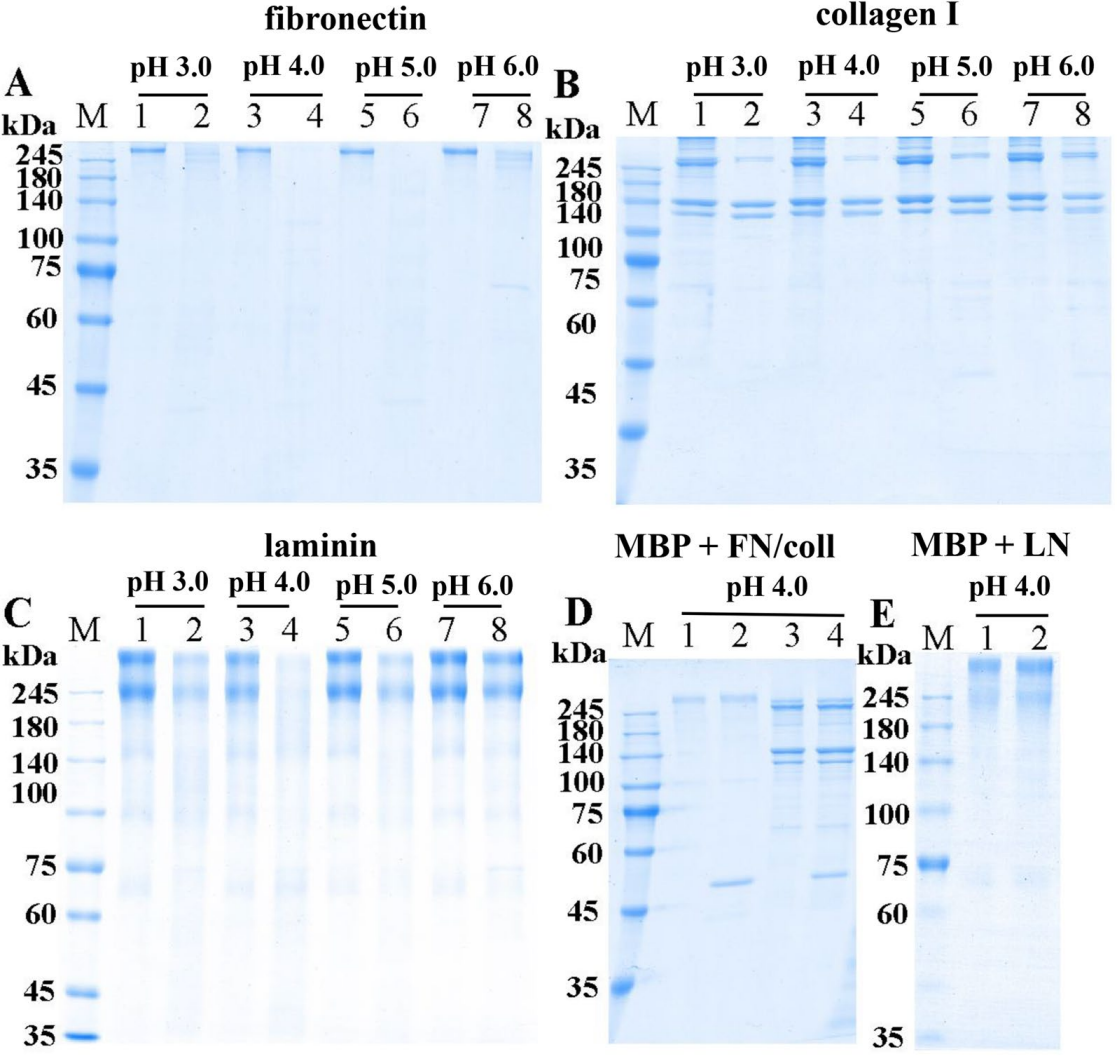
**Degradation of fibronectin, collagen I and laminin.** **A**–**C** Hydrolysis of fibronectin (**A**), collagen I (**B**) and laminin (**C**) by rTsCatL2 at different pH values. Lanes 1, 3, 5 and 7: protein; Lanes 2, 4, 6 and 8: protein + rTsCatL2. **D** Effect of MBP on hydrolysis of fibronectin and collagen I; Lane 1: fibronectin; Lane 2: fibronectin + MBP; Lane 3: collagen I; Lane 4: collagen I + MBP; (**E**) Effect of MBP on hydrolysis of laminin. Lane 1: laminin; Lane 2: laminin + MBP.

**Figure 15:**
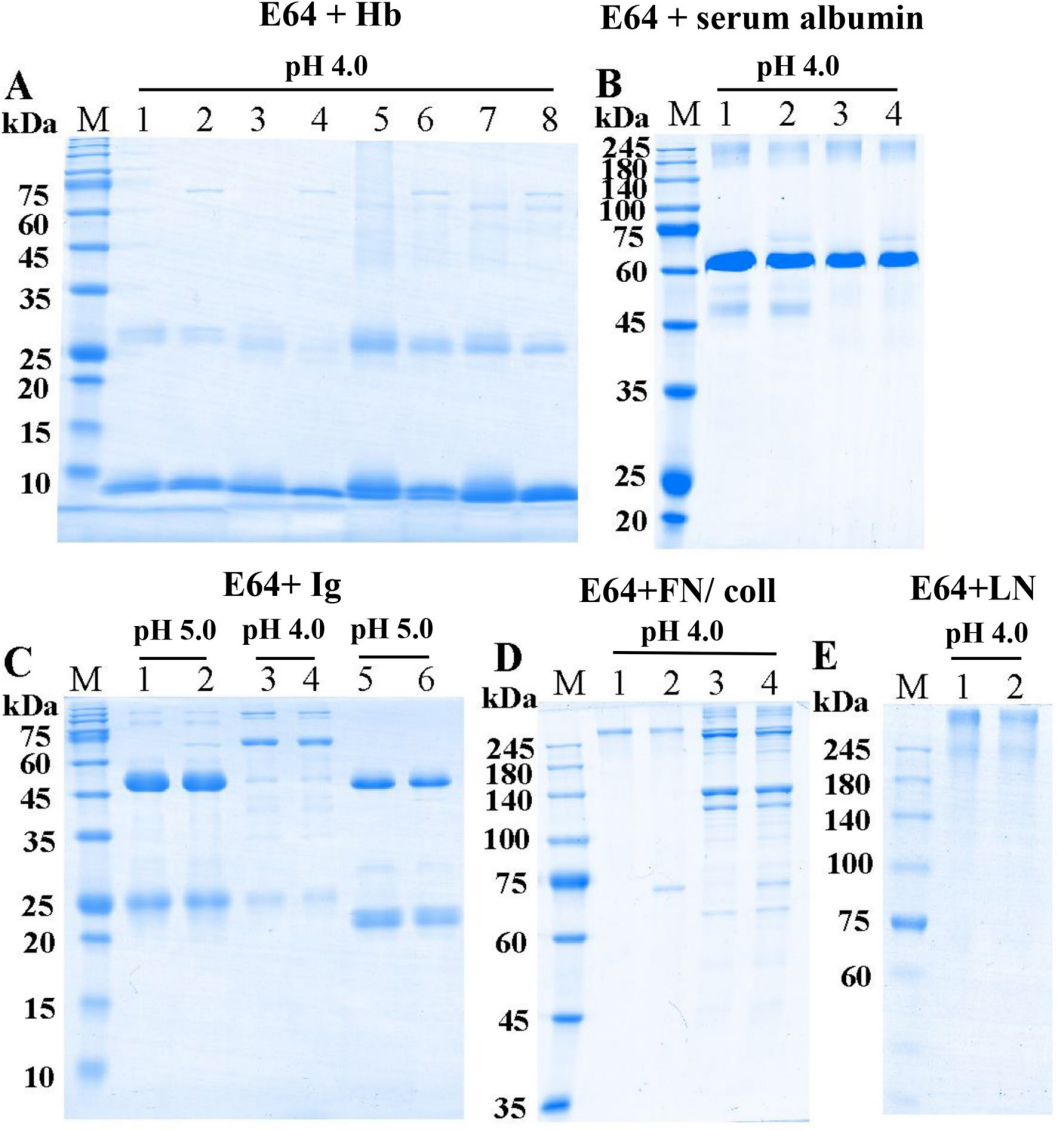
**The effects of E64 on rTsCatL2 enzymatic activity.** **A** Haemoglobin. M: protein marker, Lane 1: human Hb, Lane 2: rTsCatL2 + E64 + human Hb, Lane 3: mouse Hb, Lane 4: rTsCatL2 + E64 + mouse Hb, Lane 5: swine Hb, Lane 6: rTsCatL2 + E64 + swine Hb, Lane 7: bovine Hb, Lane 8: rTsCatL2 + E64 + bovine Hb; (**B**) BSA and HSA. Lane 1: BSA, Lane 2: rTsCatL2 + E64 + BSA, Lane 3: HSA, Lane 4: rTsCatL2 + E64 + HSA; (**C**) Immunoglobulin. Lane 1: human IgG, Lane 2: rTsCatL2 + E64 + human IgG, Lane 3: human IgM, Lane 4: rTsCatL2 + E64 + human IgM, Lane 5: mouse IgG, Lane 6: rTsCatL2 + E64 + mouse IgG; (**D**) fibronectin and collagen I. Lane 1: fibronectin, Lane 2: rTsCatL2 + E64 + fibronectin, Lane 3: collagen I, Lane 4: rTsCatL2 + E64 + collagen I; (**E**) laminin. Lane 1: laminin, Lane 2: rTsCatL2 + E64 + laminin.

The enzymatic catalysis of rTsCatL2 against several natural substrate proteins, including haemoglobin (Hb), serum albumin, immunoglobulin (Ig), fibronectin, collagen I and laminin, was assayed by SDS–PAGE. The results indicated that human, mouse, swine and bovine Hb were degraded by rTsCatL2 at pH 3.0–5.0, but degradation was not observed for chicken Hb (Figure 11). rTsCatL2 showed effective hydrolytic activity against BSA and HSA only at pH 4.0 (Figure 12). Furthermore, human IgG and mouse IgG were hydrolysed by rTsCatL2 at pH 5.0, with preferential cleavage of the heavy chain. Degradation of human IgM was detected at pH 3.0–5.0 (Figure 13). Figure 14 shows that rTsCatL2 could degrade fibronectin and collagen I at pH 3.0–6.0, with degradation of laminin at pH 3.0–5.0. Moreover, the results showed that MBP had no degradation effect on any of the proteins (Figures 11F, 12C, 13D, 14D, E). The hydrolysis of natural proteins by rTsCatL2 was completely inhibited by the addition of E64 (Figure 15).

## Discussion

Parasite cathepsin L, an important cysteine protease, is involved in the degradation of host proteins into absorbable nutrients and in the migration, development and immune evasion of the parasite in the host [13, 14]. Previous studies demonstrated that cathepsin L from *Haemonchus contortus* could degrade haemoglobin, fibrinogen, collagen, and IgG [50]. *Schistosoma mansoni* cathepsin L is localized in the gastrodermis and reproductive organs and is involved in the hydrolysis of Hb and the production of eggs [51, 52]. *Fasciola hepatica* cathepsin L was found to cleave IgG and block antibody-dependent cytotoxicity in addition to its primary role in worm invasion of tissues and degradation of nutrients [53, 54]. In *Echinococcus multilocularis*, cathepsin L was found to degrade IgG, albumin and extracellular matrix molecules [55]. A previous study indicated that *T. spiralis* cathepsin L promotes larval invasion, but this recombinant cathepsin L lacks natural cathepsin activity and cannot hydrolyse host proteins [20]. There is very limited research on *T. spiralis* cathepsin L. Since no active cathepsin L of *T. spiralis* has been previously obtained, its roles remain unknown. In this study, we successfully expressed a novel cathepsin L and confirmed its biochemical function.

Bioinformatic analysis revealed that the TsCatL protein contains a transmembrane helix, inhibitor_I29 domain and Pept_C1 domain. TsCatL has highly conserved cathepsin L active site residues (Gln, Cys, His and Asn), as well as typical ERFNIN, GYLND and GCNGG motifs, which are important for its function [56, 57]. In the phylogenetic analysis, TsCatL was localized to nematodes, which is consistent with the phylogenetic position of *T. spiralis* [58]. The 3D structures of TsCatL2 showed that the propeptide blocks the active sites of the protease, while the active sites of mature TsCatL are exposed. Molecular docking results of mature TsCatL and E64 showed hydrophobic effects and hydrogen bonding interactions, which are similar to those of *T. spiralis* cathepsins F and *Schistosoma japonicum* cathepsins B with E64 [59, 60].

Since full-length TsCatL expressed in *E. coli* has no enzymatic activity (results not shown) and the folding, coiling and disulfide bond formation of cathepsin L requires the help of the precursor peptide, the two structural domains of TsCatL were expressed in Rosetta-gamiB (DE3) cells, and the expressed protein was named TsCatL2. qPCR showed that the TsCatL gene was transcribed in the MLs, IILs, AWs and NBLs phases of *T. spiralis*, with the IILs phase showing the strongest expression and the NBLs phase showing the lowest. Western blotting showed that 60 and 56 kDa bands were recognized by anti-rTsCatL2 serum in MLs, IILs and AWs somatic proteins, but 4 bands were found in NBLs somatic proteins. The 60 kDa band likely corresponds to an inactive zymogen, and the 56 kDa band likely represents the mature active peptide. The SDS–PAGE results also indicated that rTsCatL2 can autocatalytically cleave under acidic conditions to form mature TsCatL. The inhibitor_I29 domain of TsCatL is a prepeptide that is removed by self-hydrolysis under acidic conditions, and the Pept_C1 domain becomes the mature peptide with enzymatic activity. This result is consistent with previous findings that cathepsin L proenzymes can autocatalytically cleave under acidic conditions in vitro [61–63]. Cysteine proteases usually exist as preproenzymes that can be self-hydrolysed under acidic conditions, removing the precursor peptide and transforming into an active mature enzyme [64]. The Western blot results of NBLs were inconsistent with those of other worm stages, probably because different cathepsins of *T. spiralis* are expressed as multigene families or are associated with posttranslational processing and modification of TsCatL [65, 66]. This result is consistent with other *T. spiralis* cathepsin L and *Schistosoma japonicum* cathepsin B2 proteins, which also indicated the presence of cathepsins with different molecular weights in natural worms [20, 60]. The IFA results revealed that TsCatL was located in the stichosome, gut and embryo. The gut of the parasite is an acidic environment that favours cysteine protease achievement of enzymatic hydrolytic activity [67]. In *S. mansoni*, cathepsins L1 and L2 digest haemoglobin in the digestive tract, cathepsin L2 is located in the ovovitelloduct and uterus, and its function is related to egg production [51, 52, 68]. These results suggested that TsCatL may be associated with the degradation of proteins into absorbable nutrients in the parasite gut and the development of the embryo in utero.

The enzymatic activity of rTsCatL2 was analysed by degrading the substrate Z-Phe-Arg-AMC under different conditions. The results showed that rTsCatL2 could cleave the substrate at pH 3.0–6.5, with the optimum pH of 4.5 and the optimal temperature of 55 °C. The stability of propeptide-mature enzyme interactions depends on electrostatic interactions, which may be weakened at low pH conditions, promoting conversion to the mature peptide [69]. The ability of rTsCatL2 to hydrolyse proteins was inhibited by Cu^2+^, Mn^2+^, Ni^2+^, and Zn^2+^ and enhanced by Mg^2+^. Previous studies have shown that the enzyme activities of cysteine protease from *Spirometra erinaceieuropaei* were inhibited by Cu^2+^ and Zn^2+^ [48]. Nevertheless, further studies are required to analyse the influence of metal ions on the enzymatic catalysis capacity of cathepsin L. The enzymatic activity of rTsCatL2 was significantly inhibited by E64 and PMSF and enhanced by EDTA, L-cysteine and DTT. E64 is a specific inhibitor of cysteine proteinases, PMSF inhibits serine protease and sulfhydryl protease activities, DTT is a reducing agent that prevents cross-linking of disulfide bonds, and L-cysteine is a sulfhydryl activator, which further confirms that rTsCatL2 is a sulfhydryl-containing cysteine protease [70].

Since pH is important in promoting the denaturation of various protein substrates to unfold their structures and make them more readily hydrolysed, we analysed the degradation of natural substrate proteins by rTsCatL2 at different pH values [67, 71]. Human, swine, mouse, and bovine Hb were degraded by rTsCatL2 at pH 3.0–5.0, but degradation was not observed for chicken Hb. This result implies that the cleavage of Hb by rTsCatL2 is host specific. rTsCatL2 also degrades serum albumin, and digestion of haemoglobin and serum albumin by rTsCatL2 may be related to nutrient acquisition by *T. spiralis* [13, 55, 72]. In addition, human IgG, human IgM and mouse IgG were also digested by rTsCatL2, which suggested that rTsCatL2 may help *T. spiralis* evade host immune attack by breaking down attached immunoglobulins. Cathepsin L, a protease associated with immune evasion in parasites, was found to be able to digest IgG from *Haemonchus contortus* and *Schistosoma japonicum* [50, 73]; cathepsin L1, L2 and L5 from *Fasciola hepatica* can cleave IgG [53, 72]. Many reports have shown that cysteine proteases from parasites can degrade the extracellular matrix [48, 60], and in the present study, the same function was found for *Trichinella* cathepsin L, as it degraded fibronectin, collagen I and laminin. This result suggests that cathepsin L may contribute to the invasion of *Trichinella* into the small intestinal epithelium. The degradation of all the above protein substrates by rTsCatL2 could be completely inhibited by E64. This result indicates that the hydrolysis of various substrate proteins by rTsCatL2 can be completely inhibited by E64.

In conclusion, we expressed cathepsin L of *T. spiralis* and characterized it biochemically and functionally. rTsCatL2 has the natural enzymatic activity of a cysteine protease and can degrade Hb, serum albumin, immunoglobulins, fibronectin, collagen I and laminin under acidic conditions, and its enzymatic activity is host specific. Future research could be centred on the biological functions of this TsCatL regarding the host-parasite interface in vivo and could explore a vaccine or drug against *T. spiralis* infection.

## Supplementary Information

**Additional file 1. Evaluation of the TsCatL2 3D structural model.** **A** Overall quality factor; **B** 3D-1D profile; **C** Ramachandran plot.

**Additional file 2. Evaluation of the mature TsCatL2 3D structural model.** **A** Overall quality factor; **B** 3D-1D profile; **C** Ramachandran plot.

## Abbreviations

MLs: muscle larvae
AWs: adult worms
NBLs: newborn larvae
Hb: haemoglobin
Ig: immunoglobulin
BSA: bovine serum albumin
HSA: human serum albumin
TsCatL: *T. spiralis* cathepsin L
TsCatL2: two domains of *T. spiralis* cathepsin L
rTsCatL2: recombinant TsCatL2
