# Supplementary figures and images for: Molecular characterization and determination of the biochemical properties of cathepsin L of Trichinella spiralis
Source: Vet Res. 2022 Jun 23;53:48. doi: 10.1186/s13567-022-01065-6 (PMC9229914; doi:10.1186/s13567-022-01065-6)

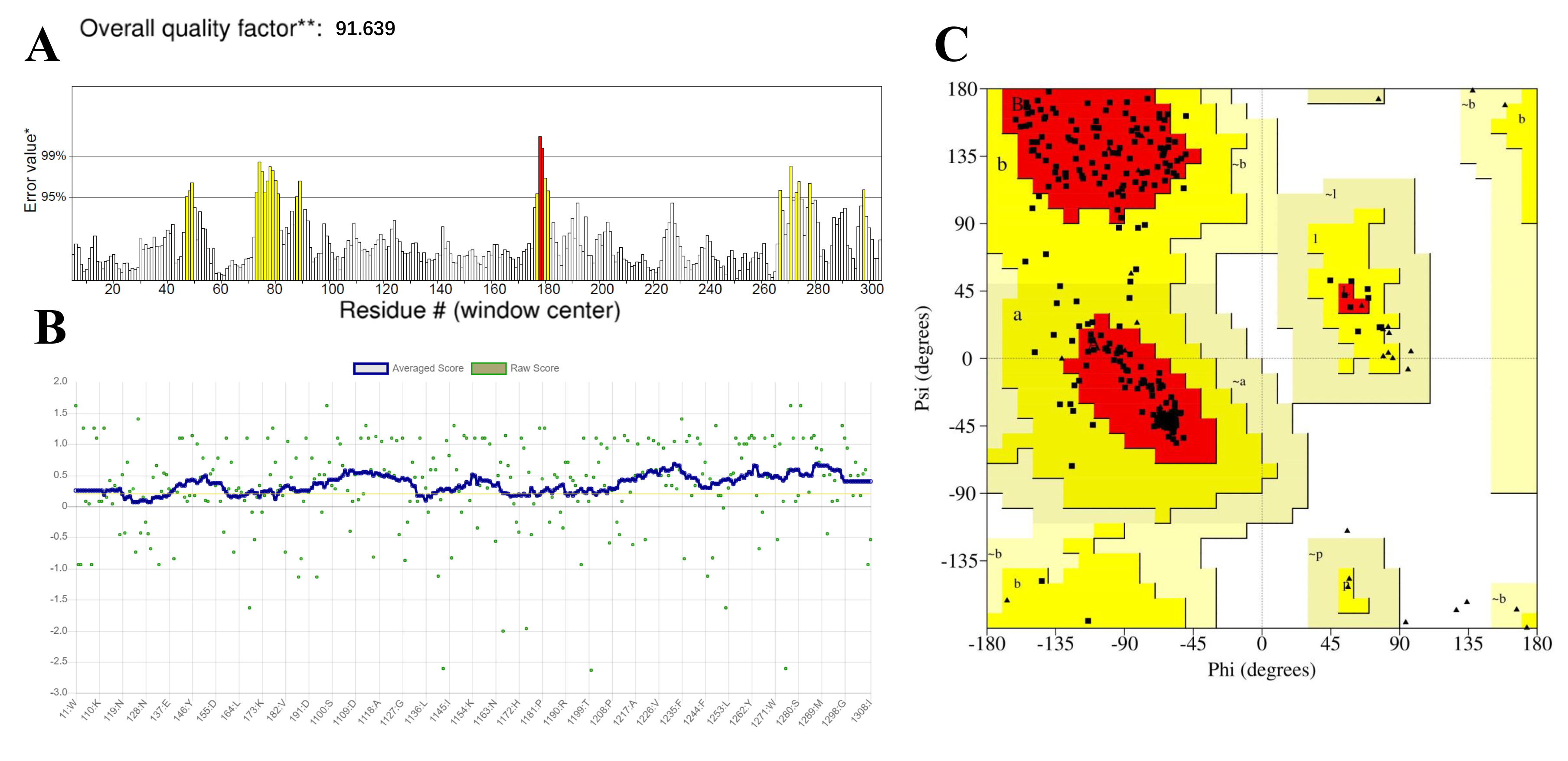

Supplement: Supplementary file 1 — Additional file 1. Evaluation of the TsCatL2 3D structural model. A Overall quality factor; B 3D-1D profile; C Ramachandran plot. [file 13567_2022_1065_MOESM1_ESM.tif]

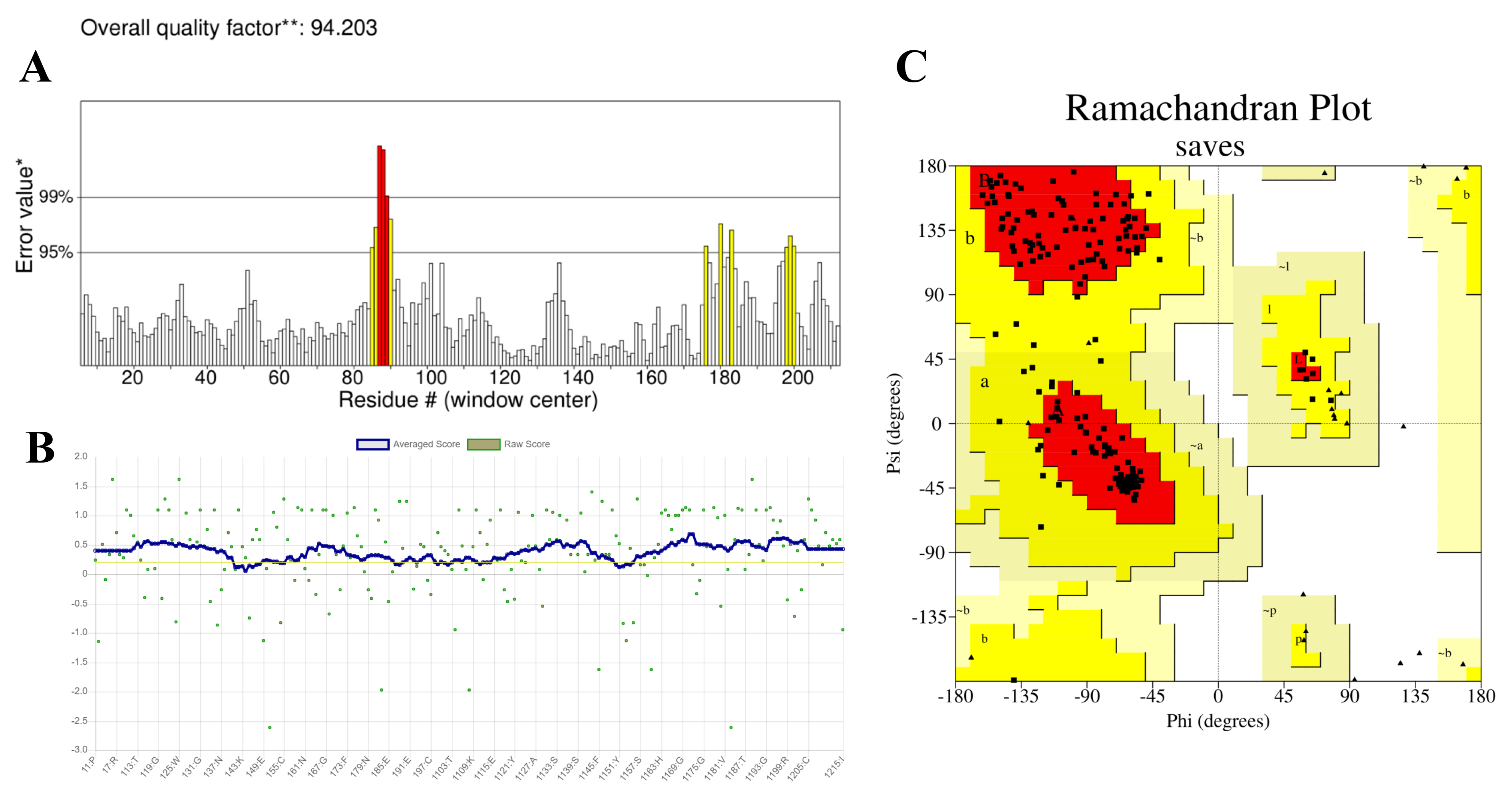

Supplement: Supplementary file 2 — Additional file 2. Evaluation of the mature TsCatL2 3D structural model. A Overall quality factor; B 3D-1D profile; C Ramachandran plot. [file 13567_2022_1065_MOESM2_ESM.tif]
